# GenReP: An Ensemble Model for Predicting TP53 in Response to Pharmaceutical Compounds

**DOI:** 10.3390/molecules31040739

**Published:** 2026-02-21

**Authors:** Austin Spadaro, Alok Sharma, Iman Dehzangi

**Affiliations:** 1 Center for Computational and Integrative Biology, Rutgers University, Camden, NJ 08102, USA; apadaro@scarletmail.rutgers.edu; 2 Institute for Integrated and Intelligent Systems, Griffith University, Brisbane, QLD 4111, Australia; alok.sharma@griffith.edu.au; 3 College of Informatics, Korea University, Seoul 02841, Republic of Korea; 4 Laboratory for Medical Science Mathematics, RIKEN Center for Integrative Medical Sciences, Yokohama 230-0045, Japan; 5 Department of Computer Science, Rutgers University, Camden, NJ 08102, USA; 6 Rutgers Cancer Institute, Rutgers University, New Brunswick, NJ 08901, USA

**Keywords:** TP53, gene expression, ensemble classifier, Connectivity Map, feature extraction

## Abstract

TP53 is a tumor-suppressor gene involved in regulating apoptosis, DNA repair, and genomic stability. Mutations in TP53 are implicated in approximately half of all detected cancers, including breast, lung, colorectal, and ovarian cancers, making it a significant target for therapeutic interventions. Many pharmaceutical drugs aim to restore TP53 function, and there is a need for predictive tools to assess how compounds may affect TP53 expression. In this study, we propose a new ensemble machine-learning model to predict the direction of TP53 relative gene expression in response to pharmaceutical compounds. Our model utilizes molecular fingerprints, descriptors, and scaffold-based features extracted from SMILES representations of compounds concatenated into a single feature vector. Trained using our newly generated benchmark dataset based on the Connectivity Map (CMap) database and addressing class imbalance with the Synthetic Minority Over-sampling Technique (SMOTE), our model achieves 62.9%, 93.9%, 40.3%, and 0.39 in terms of accuracy, sensitivity, specificity, and Matthews Correlation Coefficient (MCC), respectively. As the first-of-its-kind TP53 gene regulation prediction, our study serves as a convincing proof-of-concept that paves the way for future investigation. GenReP as a stand-alone predictor, its source code, and our newly generated benchmark dataset are publicly available.

## 1. Introduction

The TP53 gene is one of the most extensively studied tumor-suppressor genes due to its critical role in regulating cell-cycle arrest, apoptosis, and DNA repair—processes essential for preventing cancer development [1]. Mutations in TP53 are among the most common genetic alterations observed in human cancers, occurring with high frequency in various solid tumors [2]. The presence of TP53 mutations often correlates with poor prognosis, increased tumor aggressiveness, and resistance to chemotherapy, making it a key focus for both research and clinical treatment strategies [3]. Additionally, mutant TP53 can acquire gain-of-function (GOF) properties that contribute to tumor progression and therapeutic resistance, emphasizing the importance of understanding TP53 regulation in cancer biology [4].

Accurately predicting gene-expression responses to drug treatments is critical for advancing personalized medicine and cancer therapy [5]. Understanding how pharmaceutical compounds modulate TP53 expression provides valuable insights for tailoring treatment strategies, particularly in cancers where TP53 mutations drive resistance to standard therapies [6]. This predictive capability also holds promise for drug development, enabling the prioritization of compounds that exhibit favorable gene regulatory effects and ultimately improving therapeutic outcomes [7].

During the past few years, several computational approaches have been developed to predict gene-expression responses to various stimuli. For example, Alipanahi et al. introduced DeepBind, a deep-learning model using convolutional neural networks (CNNs) to predict DNA- and RNA-binding protein sequence specificities, demonstrating how machine learning could decipher complex patterns in genomic sequences that influence gene expression [8]. Similarly, Zhou and Troyanskaya developed DeepSEA, a deep-learning approach that predicts the chromatin effects of noncoding variants by modeling high-order interactions within the genome [9]. More recently, Kuenzi et al. introduced a deep-learning model that predicts gene-expression responses to drug treatments using compound structural features, highlighting the potential of deep learning in pharmacogenomics [10].

Further works present the application of transformer-based models to molecular representation learning from Simplified Molecular Input Line Entry System (SMILES) strings for various tasks such as molecular property prediction and molecule generation. For example, Wang et al. introduced SMILES-BERT, a transformer-based model pre-trained on large chemical databases to predict molecular properties directly from SMILES representations [11]. Similarly, Chithrananda et al. developed ChemBERTa, which leverages self-supervised pre-training on large-scale chemical data for improved molecular property prediction [12]. Li and Jiang proposed Mol-BERT, utilizing the BERT architecture to handle SMILES representations for predicting molecular properties and chemical reactions [13].

However, these studies primarily focus on predicting gene-expression changes due to genetic variations, chemical properties, or general regulatory mechanisms rather than responses to specific pharmaceutical compounds targeting TP53. To the best of our knowledge, this is the first study that develops a machine-learning model specifically aimed at predicting TP53 expression changes in response to drug treatments.

In this study, we leverage the Connectivity Map (CMap) database, a comprehensive resource that catalogs gene-expression data in response to a wide array of drug treatments, generate a new benchmark dataset for TP53 gene regulation prediction. To the best of our knowledge, this dataset will also be of the first of its kind for TP53 gene regulation prediction. We use this dataset to train a new machine-learning model, called GenReP, capable of predicting the regulatory response of the TP53 gene to a given pharmaceutical compound [14,15]. By incorporating molecular descriptors, chemical fingerprints, and structural features, our ensemble model classifies TP53 expression as upregulated, downregulated, or unregulated.

GenReP achieves an accuracy of 62.9%, sensitivity of 93.9%, specificity of 40.3%, and a Matthews Correlation Coefficient (MCC) of 0.39 in binary classification. In multi-class classification, it attains an overall accuracy of 58.3% and an average sensitivity, specificity, and MCC of 46.6%, 79.1%, and 0.25. Our results demonstrate the model’s potential to improve therapeutic decision-making by offering new insights into drug-gene interactions and advancing cancer treatment strategies. GenReP as a stand-alone predictor, its source code, and our generated benchmark dataset are publicly available at https://github.com/MLBC-lab (accessed on 12 February 2026).

## 2. Results and Discussion

In this section, we discuss the evaluation metrics, methods, and results used in the study to predict TP53 regulation. We also examine the performance of the features employed in the model, highlighting their contribution to the overall prediction effectiveness.

### 2.1. Evaluation Metrics

The performance of our classification models is evaluated using several metrics, including accuracy, specificity, sensitivity, and MCC [16,17,18]. These metrics are defined by the following equations:

(1) $$sensitivity=\frac{TP}{TP+FN}$$

(2) $$specificity=\frac{TN}{TN+FP}$$

(3) $$accuracy=\frac{TP+TN}{TP+FP+TN+FN}$$

(4) $$MCC=\frac{\left(TN\times TP\right)-\left(FN\times \;FP\right)}{\sqrt{(TP+FP)\;\left(TP+FN\right)(TN+FP)\;(TN+FN})}$$

In these formulas, TP represents true positives, which is the number of correctly identified positive instances, TN represents true negatives or the number of correctly identified negative instances. FP represents false positives, which is the number of incorrectly classified negative samples as positive, and FN which represents false negatives, which is the number of positive instances that are incorrectly identified as negative.

Accuracy, sensitivity, and specificity range from 0% (indicating all predictions are incorrect) to 100% (indicating all predictions are correct). The MCC ranges from −1 to 1, with 0 indicating predictions equivalent to random guessing. In this section, the abbreviations ACC, SP, SN, and MCC are used to represent Accuracy, Specificity, Sensitivity, and MCC, respectively.

### 2.2. Evaluation Methods

To assess the model’s performance, both k-fold cross-validation and an independent test set are used [19]. In k-fold cross-validation, the dataset is divided into *k* partitions. For each iteration, *k* − 1 partitions are used for training, while the remaining partition serves as the validation set [20]. This process repeats *k* times, ensuring that each partition is used for validation once. The results from each iteration are averaged to produce a final score. In this study, k-fold values of 5 and 10 were used. For the independent test set, 10% of the data is reserved for validation, while 90% is used for training [21,22]. The ratio of positive to negative samples in the test set matches the imbalance found in the training set [23].

### 2.3. Ensemble Classification Results

**Figure 1:**
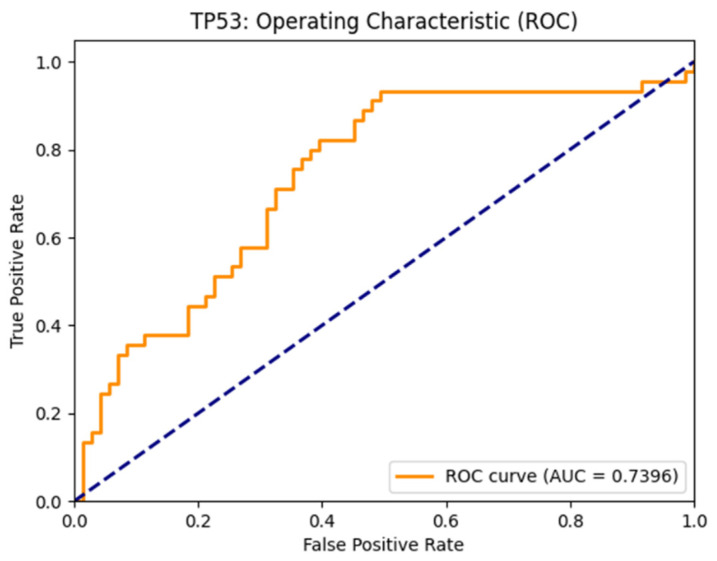
Binary classifier receiver-operating characteristic curve.

To evaluate the performance of GenReP, we experiment with two types of classification tasks: binary classification and multiclass classification. In the binary classification task, we focus on drugs known to affect TP53 expression—that is, drugs that either upregulate or downregulate the TP53 gene. We aim to predict whether each drug will result in upregulation or downregulation of TP53 expression. Thus, GenReP distinguishes between two classes: “upregulated” and “downregulated.” To evaluate this model, we withhold 10% of the available data for testing. Using this independent test set, our binary classification model achieves an accuracy of 62.9%, sensitivity of 93.9%, specificity of 40.3%, and a MCC of 0.39. This model achieves an AUC score of 0.74 [24,25,26]. The Receiver-Operating Characteristic Curve (ROC) for this model is presented in Figure 1.

In the multiclass classification task, we expand our scope to include all drugs in the dataset, including those that do not significantly affect TP53 expression. Here, the model predicts whether a drug will result in the TP53 gene being upregulated, downregulated, or not regulated. This task involves three classes:

**Upregulated**: Drugs that increase TP53 expression (Z-score > +1).

**Downregulated**: Drugs that decrease TP53 expression (Z-score < −1).

**Not regulated**: Drugs that do not significantly change TP53 expression (−1 ≤ Z-score ≤ +1).

Evaluating GenReP against the 10% holdout set, we achieve an overall accuracy of 58.3%. The individual class accuracies are 75.7% for upregulated, 75.3% for downregulated, and 65.5% for not regulated. These results are presented in Table 1. We also evaluate our multiclass model using 5-fold and 10-fold cross-validation. For both 5-fold and 10-fold cross-validation we use Stratified K-Fold and apply SMOTE only to the training split of each fold (the validation split is never augmented). Concretely, for each fold we fit SMOTE on the training data, synthesize minority-class samples, train the ensemble, and evaluate on the untouched validation split. The independent 10% hold-out set is also never augmented. This protocol addresses class imbalance within training while preventing leakage. The results of the 5-fold and 10-fold cross-validation are presented in Table 2 and Table 3, respectively.

As shown in Table 3, performance remains lower for the multiclass task than for the binary task, as the model must decide both whether TP53 is perturbed and in which direction. Under this protocol, upregulated and downregulated sensitivities reach the low–mid-40% range in 5- and 10-fold CV, while specificities remain near ~77–79%, closely matching the independent test set. Per-class MCC falls around ~0.24–0.31 for the minority classes and ~0.36–0.38 for not-regulated, indicating balanced performance across categories.

Cross-validation results prioritize recall for the up/down classes with a small, expected reduction in specificity, yielding the MCC values shown in Table 2 and Table 3. Macro-level CV metrics track the independent test set closely, suggesting that the in-fold SMOTE protocol mitigates class imbalance without leakage and provides a reliable estimate of generalization.

For the binary task, the model retains high sensitivity under the same in-fold SMOTE procedure, aligning with the practical goal of minimizing false negatives when flagging potentially active compounds.

Although overall multiclass accuracy remains moderate, the stronger minority-class sensitivity and solid MCC indicate a practically balanced ensemble once class imbalance is addressed during training. As the first method explicitly targeting TP53 regulation direction from compound structure alone, our study shows that machine-learning can automate this decision with informative reliability. Future work will explore richer molecular encodings (e.g., 3D/graph features, scaffold-aware tokenization, large-scale chemical pretraining), calibrated decision thresholds/cost-sensitive learning, and transformer-based architectures to further enhance direction-specific prediction under class imbalance.

### 2.4. Ensemble Evaluation

**Figure 2:**
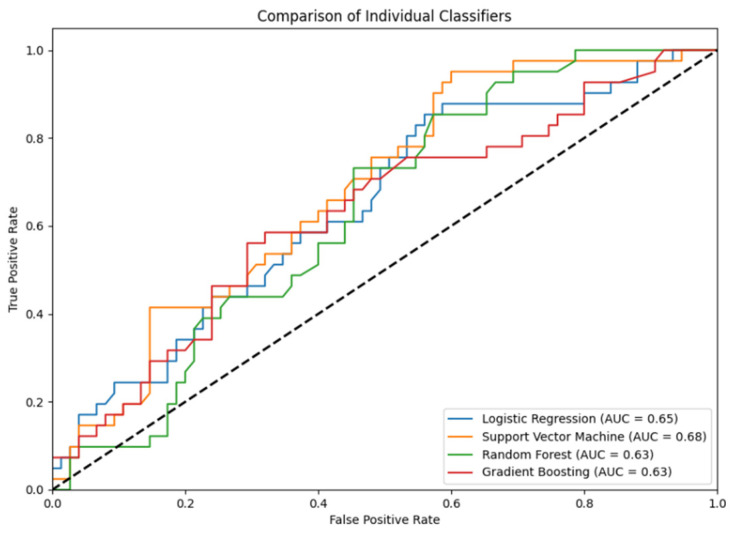
ROC comparison between separate classifiers.

Ensemble classification combines multiple machine-learning algorithms to improve predictive performance by leveraging the strengths of each individual model and reducing overfitting [27,28,29,30]. In developing our model, we considered various algorithms—including Logistic Regression (LR), Support Vector Machine (SVM), Random Forest (RF), Gradient Boosting Classifier (GBC), and others—but found that integrating LR, SVM, RF, and GBC using a soft voting mechanism yielded the best results. While individual classifiers performed comparably when tested independently, the ensemble model GenReP consistently outperformed them, as shown in Figure 2, which illustrates the Area Under the Receiver Operating Characteristic Curve (AUC) for the individual classifiers.

### 2.5. Feature Evaluation

In this section, we evaluate the contribution of each feature set to ensure that they all contribute to the model’s performance. Too many features may introduce unnecessary noise into the model, so we demonstrate that each set is necessary [31,32,33]. To do this, we iteratively remove each feature set from the training data and independently evaluate the model against the 10% independent test set. For this comparison, we use our highest performing model, which is binary classification. The results of this evaluation are presented in Table 4. The biggest impact seen in this experiment is removing the Morgan fingerprints, which drastically reduces accuracy to 51.2%, indicating it is the most informative feature set. Removing the molecular descriptor and scaffold features lowered accuracy to 61.50% and 61.8%, respectively. While these drops are much less significant, they do indicate that they contribute to the model and are necessary.

To summarize, the main key takeaways of this study can be presented as follows:

Across both evaluation protocols, GenReP demonstrates that chemical structure contains a measurable but incomplete signal for TP53 directionality. Performance is strongest in the binary setting, which can be useful for prioritization. The multiclass setting remains challenging, especially for the upregulated class, suggesting that heterogeneous biological context and label ambiguity near the threshold are likely to limit prediction quality from structure alone. A future direction may be to expand this approach to also account for biologically relevant factors, such as cell type, dose, exposure time, etc. These results, as they are, motivate using GenReP as a screening tool rather than a definitive decision system.

## 3. Materials and Methods

This section details the proposed method and database used in this study.

### 3.1. Benchmark Dataset

#### 3.1.1. The Connectivity Map (CMap)

CMap is an extensive resource developed by the Broad Institute MIT and Harvard University to explore the molecular and cellular effects of a variety of chemical compounds, genetic perturbations, and biological stimuli [14]. As part of the NIH Library of Integrated Network-Based Cellular Signatures (LINCS) initiative, CMap provides researchers with access to more than 1.3 million gene expression profiles generated using a high-throughput platform called L1000 [15]. This database aims to uncover connections between drugs, genes, and disease states based on common gene-expression signatures, facilitating research in drug discovery, functional genomics, and precision medicine.

#### 3.1.2. The L1000 Assay

The L1000 assay is a reduced representation expression profiling method that measures the expression levels of 978 selected “landmark” genes. These genes are chosen because they capture the majority of variation in the transcriptome and are used to infer the expression of approximately 80% of the remaining genes. L1000 uses ligation-mediated amplification followed by hybridization to polystyrene microspheres, each designed to detect specific transcripts via fluorescence [15]. This method results in a highly scalable, cost-effective approach for generating gene expression profiles that are comparable to RNA sequencing in accuracy. The data are typically represented as Z-scores, quantifying the relative expression changes in each gene in response to a treatment compared to a control.

#### 3.1.3. Data Representation and Accessibility

In addition to chemical perturbations, CMap contains data on genetic perturbations, such as knockdowns and over-expressions performed on a broad range of cell lines. This allows researchers to explore the direct effects of small molecules on gene expression as well as how genes interact within various biological pathways. The CMap dataset is accessible through an interactive platform called CLUE (Connectivity Map Linked User Environment), enabling users to explore gene-expression signatures and identify mechanistic insights, potential drug targets, and off-target effects of pharmaceutical compounds. This platform and data are publicly available at https://clue.io/ (accessed date: 12 February 2026).

#### 3.1.4. Extraction of TP53 Gene Expression Data

For this study, we extract the gene-expression data specifically for the TP53 gene from the CMap database, focusing on its response to a wide range of pharmaceutical compounds. This TP53-specific data allows us to train a new machine-learning model that predicts whether a given drug will upregulate, downregulate, or have no significant effect on TP53 expression. By utilizing the comprehensive data from CMap, we provide new insights into the regulatory mechanisms of TP53 in response to pharmaceutical perturbations.

**Figure 3:**
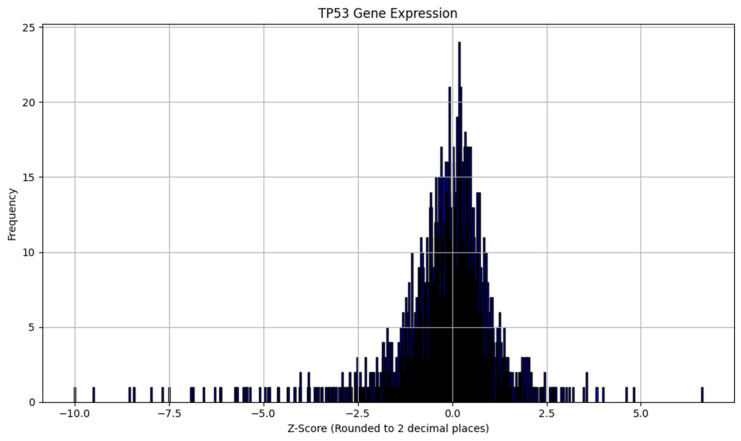
Frequency of extracted TP53 z-scores.

**Figure 4:**
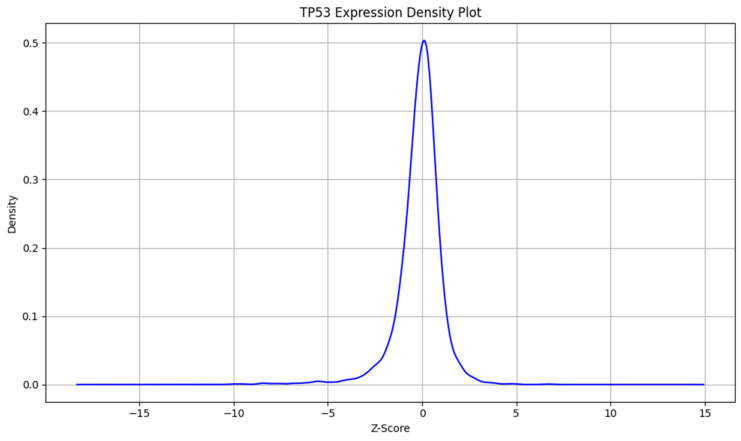
Density plot of extracted TP53 z-scores.

To understand the distribution of TP53 gene-expression changes in our dataset, we present a frequency plot in Figure 3 and a density plot in Figure 4 of the extracted Z-scores. The frequency plot displays the number of occurrences of TP53 expression changes within specified Z-score intervals, illustrating how often certain levels of gene expression changes occur. The density plot provides a smoothed visualization of the data, estimating the probability density function of the Z-scores to highlight the overall shape of the distribution. Both plots reveal that the majority of TP53 Z-scores cluster around zero, indicating that most compounds have little to no significant effect on TP53 expression. This suggests a high prevalence of the “not regulated” class in our dataset and underscores the class imbalance, with fewer compounds causing substantial upregulation or downregulation.

#### 3.1.5. Time-Course Data and Peak Response Analysis

Some pharmaceutical compounds in CMap are tested at multiple intervals to capture how gene expression changes after exposure [14]. Gene-expression responses to a drug typically follow a dynamic trajectory in which initially, expression increases as the cell responds to the compound, eventually reaching a peak response [34,35,36]. After this peak, expression levels recede, gradually returning to baseline values as the drug’s effects diminish. We analyze the peak response from the time-course data to ensure that each compound’s most significant gene expression changes are used. By focusing on the peak expression, this approach captures the maximal regulatory effect of each drug on the TP53 gene, providing a clearer picture of the gene’s response to pharmaceutical treatments [37].

#### 3.1.6. Classification of TP53 Gene Expression Changes

**Figure 5:**
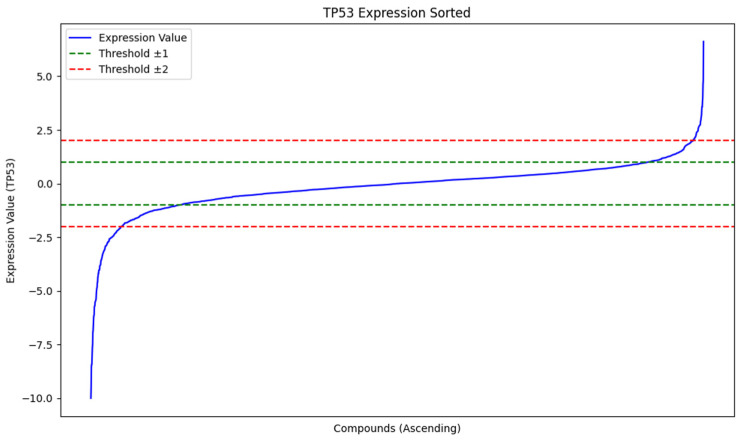
Sorted TP53 expression responses across benchmark compound perturbations.

To classify TP53 gene-expression changes as upregulated, downregulated, or not regulated, we use the Z-scores obtained from the L1000 assay. The Z-score represents the number of standard deviations by which the expression level of TP53 deviates from the mean expression level under control conditions [14,15]. A positive Z-score indicates that TP53 expression is higher than the control mean (upregulation), while a negative Z-score indicates it is lower (downregulation) [38]. Since Z-scores are continuous variables, we apply thresholds to discretize them into categorical classes [39]. We consider thresholds at ±1 and ±2 standard deviations; however, we observe that an insignificant number of samples exceed the ±2 threshold, making it impractical for effective classification. Therefore, we adopt the ±1 threshold for this study. Specifically, we classify TP53 as upregulated if the Z-score is greater than +1, downregulated if the Z-score is less than −1, and not regulated if the Z-score falls between −1 and +1. These thresholds are visualized and presented in Figure 5. The number of classifications for each threshold is presented in Table 5.

### 3.2. Data Augmentation

We experimented with both binary and multi-class classification to predict TP53 regulation outcomes. For multi-class classification, we predict whether a drug will result in the TP53 gene becoming upregulated, downregulated, or not regulated. Since there are far more gene–drug interactions in our dataset that do not result in TP53 regulation, there is potential for the model to bias toward these neutral samples [40,41,42]. To address this class imbalance and prevent bias toward the more prevalent neutral samples, we apply the Synthetic Minority Over-sampling Technique (SMOTE) for data augmentation [43,44,45] to increase the number of both up- and downregulated samples. Because the ratio of down- to upregulated samples is also imbalanced, SMOTE was also used to increase the number of downregulated samples when performing binary classification.

SMOTE generates synthetic samples for the minority classes by interpolating between existing instances, increasing the representation of the underrepresented classes (upregulation and downregulation) without simply duplicating existing examples. SMOTE is commonly used for addressing data imbalances and has been shown to be effective for chemical and SMILES data [46,47,48,49]. We separate the data used for our independent test set before augmentation to avoid overfitting. All augmented data are used only for training and not for evaluation.

### 3.3. Feature Representation

Accurate feature representation is critical in developing an effective machine-learning model for predicting biological responses to pharmaceutical compounds. We derive features directly from the SMILES strings of chemical compounds, a standard textual representation of molecular structures [50,51,52]. SMILES encodes molecular structures as linear strings by specifying atoms, bond connectivity, and stereochemistry. It captures chemical information such as atomic identities, the types of bonds between atoms (single, double, triple), ring structures, and chirality in a human-readable format. This representation allows the conversion of molecular structures into numerical features systematically, enabling machine-learning models to interpret and learn from the underlying chemical and structural properties of the compounds.

By converting these SMILES strings into numerical features, we enable the machine-learning model to interpret and learn from the structural and chemical properties of the compounds. Our feature extraction process encompasses three primary types of features, namely molecular fingerprints, molecular descriptors, and scaffold-based features which are introduced in more detail in the following subsections. Each type captures different aspects of the chemical compounds, providing a comprehensive representation that enhances the predictive capability of the model [53,54,55].

#### 3.3.1. Molecular Fingerprints

Molecular fingerprints are binary vectors that encode the presence or absence of substructures within a molecule. Widely used in cheminformatics for similarity searching and quantitative structure–activity relationship (QSAR) modeling tasks, we utilize the Morgan fingerprint algorithm with a radius of 2, generating a 2048-bit vector for each compound [53]. The Morgan fingerprint is a circular fingerprinting method that considers atom neighborhoods within a specified radius, effectively capturing the local structural features of the molecule. Morgan fingerprinting is used extensively in cheminformatic studies and proves to be an effective strategy for chemical feature representation [56,57,58,59,60].

#### 3.3.2. Molecular Descriptors

Molecular descriptors are numerical values that quantify various physicochemical properties of a molecule, providing insights into characteristics that may affect biological activity [54,61]. We calculate the following molecular descriptors for each compound using the RDKit cheminformatics library [62]:

**Molecular Weight (MW)**: The sum of the atomic weights of all atoms in the molecule, influencing the compound’s absorption, distribution, metabolism, and excretion (ADME) properties [63].

**LogP**: The logarithm of the partition coefficient between n-octanol and water, indicating the compound’s hydrophobicity, affecting membrane permeability and solubility [64].

**Number of Rotatable Bonds (NRB)**: Counts the bonds that allow free rotation, excluding certain bond types associated with molecular flexibility influencing binding interactions [65].

**Number of Hydrogen Bond Donors (HBD)**: The total number of hydrogen atoms attached to electronegative atoms like oxygen or nitrogen, crucial for molecular recognition processes [66].

**Number of Hydrogen Bond Acceptors (HBA)**: The total number of electronegative atoms with lone pairs that can accept hydrogen bonds, playing a significant role in intermolecular interactions [66].

By incorporating these descriptors, our model gains quantitative information about properties known to influence drug behavior and interactions with biological targets [67].

#### 3.3.3. Scaffold-Based Features

Scaffolds represent the core structures of molecules, capturing the fundamental skeleton upon which functional groups are attached [55]. They are essential for understanding structure–activity relationships because they highlight common frameworks shared among bioactive compounds [68].

**Figure 6:**
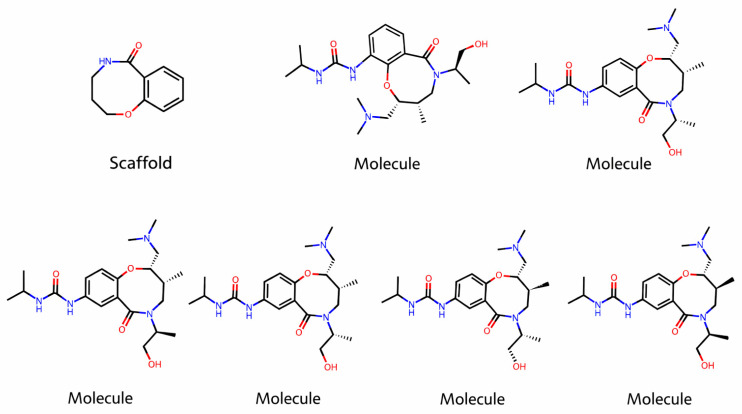
An example scaffold identified in our dataset.

We extract the Bemis–Murcko scaffolds from each molecule using RDKit’s scaffold module. The Bemis–Murcko scaffold is defined by the ring systems and the linkers connecting them, effectively stripping away side chains and focusing on the core structure [69]. An example scaffold identified in our dataset is presented in Figure 6. We compile a list of unique scaffolds present in our dataset and create binary features indicating the presence or absence of each scaffold in a given molecule.

This approach allows the model to recognize whether a compound contains a scaffold commonly associated with upregulation or downregulation of TP53 expression. By encoding scaffold information, we capture higher-level structural patterns that might not be evident through fingerprints or basic descriptors alone [70].

#### 3.3.4. Feature Vector Composition

For each compound, we concatenate the molecular fingerprint, molecular descriptors, and scaffold-based features to form a comprehensive feature vector. Specifically, the feature vector for each molecule consists of the following:

**Molecular Fingerprint**: A 2048-bit binary vector from the Morgan fingerprint.

**Molecular Descriptors**: A 5-element numerical vector containing MW, LogP, NRB, HBD, and HBA.

**Scaffold-Based Features**: A binary vector of length *n*, where *n* is the number of unique scaffolds in the dataset, indicating the presence (1) or absence (0) of each scaffold in the molecule.

### 3.4. Classification Technique

**Figure 7:**
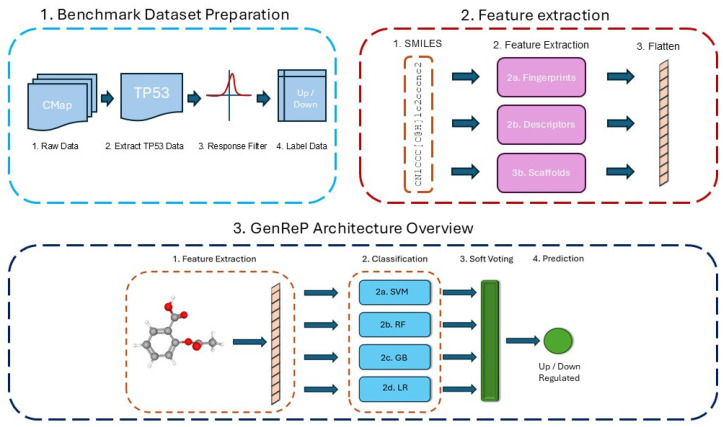
The general pipeline of our study to build GenReP. This figure illustrates the three main stages of our approach. First, the Benchmark Dataset Preparation (**box 1**) involves extracting TP53 gene expression data from the CMap database, applying a response filter based on the L1000 assay, and labeling samples. Second, the Feature Extraction (**box 2**) step converts each compound’s SMILES representation into numerical features, including molecular fingerprints, physicochemical descriptors, and scaffold-based features. Finally, the GenReP Architecture Overview (**box 3**) integrates these features into an ensemble model comprising four classifiers (SVM, RF, GB, and LR). The final prediction of TP53 regulation is the result of soft voting between the four classifiers.

We employ an ensemble classification model consisting of multiple machine-learning algorithms to predict the regulatory response of the TP53 gene to pharmaceutical compounds [71,72,73]. Our ensemble integrates four classifiers: Logistic Regression (LR), Support Vector Machine (SVM), Random Forest (RF), and Gradient Boosting Classifier (GBC) [74,75,76,77,78]. These models are combined using a soft voting mechanism, where the predicted class is determined by averaging the probability scores from each classifier [79,80]. This approach allows the model to leverage the strengths of each classifier and provide a more robust prediction. Our model’s schematic flowchart, including the GenReP architecture, is illustrated in Figure 7.

## 4. Conclusions

In this study, we developed a new machine-learning model, called GenReP, to predict the regulatory response of the TP53 gene to pharmaceutical compounds using our newly generated benchmark dataset derived from the CMap database. Our ensemble model integrates Logistic Regression, Support Vector Machine, Random Forest, and Gradient Boosting classifiers, leveraging molecular fingerprints, descriptors, and scaffold-based features extracted from SMILES strings. An analysis of each feature set confirmed that they are all necessary for our model. The model achieved 62.9% accuracy in binary classification and 58.3% in multiclass classification. We validated the model using 5-fold and 10-fold cross-validation, as well as an independent test set. The use of SMOTE for data augmentation improved performance in addressing class imbalance, particularly for underrepresented classes. These results serve as a proof-of-concept for the potential of our approach for predicting drug-induced gene regulation and contribute to advancing computational methods in drug–gene interaction research. We have made our new benchmark dataset, publicly available at https://people.camden.rutgers.edu/dehzangi-lab/files/TP53_Data.zip (access date: 12 February 2026). Additionally, GenReP, as a stand-alone predictor, and its complete source code (including the feature extraction process) can be found at https://github.com/MLBC-lab/GenReP (access date: 12 February 2026).

## Figures and Tables

**Table 1:** Independent test set results for multiclass classification.

	Accuracy	Sensitivity	Specificity	MCC
Upregulated	75.7%	18.1%	78.9%	0.17
Downregulated	75.3%	31.4%	79.5%	0.25
Not-Regulated	65.5%	90.3%	79.0%	0.34

**Table 2:** The results achieved for five-fold multiclass cross-validation.

	Accuracy	Sensitivity	Specificity	MCC
Upregulated	74.0%	41.0%	78.0%	0.24
Downregulated	73.6%	43.5%	79.0%	0.29
Not-Regulated	66.3%	87.0%	77.0%	0.36

**Table 3:** The results achieved for 10-fold multiclass cross-validation.

	Accuracy	Sensitivity	Specificity	MCC
Upregulated	75.0%	43.0%	78.6%	0.27
Downregulated	74.4%	44.9%	79.4%	0.31
Not-Regulated	67.1%	88.0%	77.5%	0.38

**Table 4:** Impact of removing each individual feature set.

	Accuracy	Sensitivity	Specificity	MCC
All Combined	62.9%	93.9%	40.3%	0.39
Morgan Fingerprints	51.2%	52.3%	50.1%	0.02
Molecular Descriptors	61.5%	92.0%	39.5%	0.37
Scaffold Features	61.8%	92.5%	39.8%	0.38

**Table 5:** The number of each classification for each threshold.

	Threshold (−1, 1)	Threshold (−2, 2)
Up Regulated	9461	2395
Downregulated	1267	117
Not Regulated	21,555	29,771

## Data Availability

Our new benchmark dataset is publicly available at https://people.camden.rutgers.edu/dehzangi-lab/files/TP53_Data.zip (accessed on 12 November 2025). Additionally, GenReP as a stand-alone predictor, and its complete source code are publicly available at: https://github.com/MLBC-lab/GenReP (accessed on 12 November 2025).
